# Diagnostic accuracy of preoperative magnetic resonance imaging for detecting subscapularis tendon tears: a diagnostic test study

**DOI:** 10.1590/1516-3180.2020.014605062020

**Published:** 2020-08-21

**Authors:** Lucas Busnardo Ramadan, Eduardo Baptista, Felipe Ferreira de Souza, Mauro Emilio Conforto Gracitelli, Jorge Henrique Assunção, Fernando Brandao Andrade-Silva, Arnaldo Amado Ferreira-Neto, Eduardo Angeli Malavolta

**Affiliations:** I MD. Attending Orthopedic Surgeon, Hospital das Clinicas HCFMUSP, Faculdade de Medicina, Universidade de Sao Paulo, Sao Paulo, SP, BR.; II MD, PhD. Attending Musculoskeletal Radiologist, Hospital das Clinicas HCFMUSP, Faculdade de Medicina, Universidade de Sao Paulo, Sao Paulo, SP, BR; Attending Musculoskeletal Radiologist, Imaging Department, Musculoskeletal Radiology Division, Hospital Israelita Albert Einstein (HIAE), São Paulo, Brazil.; III MD. Attending Musculoskeletal Radiologist, Hospital das Clinicas HCFMUSP, Faculdade de Medicina, Universidade de Sao Paulo, Sao Paulo, SP, BR.; IV MD, PhD. Attending Orthopedic Surgeon, Hospital das Clinicas HCFMUSP, Faculdade de Medicina, Universidade de Sao Paulo, Sao Paulo, SP, BR.; V MD. Attending Orthopedic Surgeon, Hospital das Clinicas HCFMUSP, Faculdade de Medicina, Universidade de Sao Paulo, Sao Paulo, SP, BR.; VI MD, PhD. Attending Orthopedic Surgeon, Hospital das Clinicas HCFMUSP, Faculdade de Medicina, Universidade de Sao Paulo, Sao Paulo, SP, BR.; VII MD, PhD. Head of the Shoulder and Elbow Group, Orthopedic Department, Hospital das Clinicas HCFMUSP, Faculdade de Medicina, Universidade de Sao Paulo, Sao Paulo, SP, BR.; VIII MD, PhD. Attending Orthopedic Surgeon, Hospital das Clinicas HCFMUSP, Faculdade de Medicina, Universidade de Sao Paulo, Sao Paulo, SP, BR.

**Keywords:** Rotator cuff, Magnetic resonance imaging, Arthroscopy, Diagnosis, Subscapularis tendon, Accuracy, Inter-observer agreement, Intra-observer agreement

## Abstract

**BACKGROUND:**

The accuracy of magnetic resonance imaging (MRI) for making the diagnosis of subscapularis tears presents wide variation in the literature and there are few prospective studies.

**OBJECTIVE:**

To compare the findings from MRI and arthroscopy for diagnosing subscapularis tears.

**DESIGN AND SETTING:**

Diagnostic test study performed in a tertiary care hospital.

**METHODS:**

We included patients who underwent arthroscopic rotator cuff repair and who had firstly undergone high magnetic field MRI without contrast. The images were independently evaluated by a shoulder surgeon and two musculoskeletal radiologists. Sensitivity, specificity, positive and negative predictive values, accuracy and inter and intra-observer agreement were calculated.

**RESULTS:**

MRIs on 200 shoulders were evaluated. The incidence of subscapularis tears was 69.5% (41.5% partial and 28.0% full-thickness). The inter and intra-observer agreement was moderate for detection of subscapularis tears. The shoulder surgeon presented sensitivity of 51.1% to 59.0% and specificity of 91.7% to 94.4%. The radiologists showed sensitivity of 83.5% to 87.1% and specificity of 41% to 45.9%. Accuracy ranged from 60.5% to 73.0%.

**CONCLUSION:**

The 1.5-T MRIs without contrast showed mean sensitivity of 70.2% and mean specificity of 61.9% for detection of subscapularis tears. Sensitivity was higher for the musculoskeletal radiologists, while specificity was higher for the shoulder surgeon. The mean accuracy was 67.6%, i.e. lower than that of rotator cuff tears overall.

## INTRODUCTION

Although the biomechanical importance of the subscapularis tendon has been recognized in biomechanical^1^ and clinical studies,^2^ it has long received little attention in the medical literature,^3^ and has been called the “forgotten tendon”.^4^ Only 1% of rotator cuff tears affect only the subscapularis,^5^^,^^6^ but more than half of all patients with supraspinatus tears present an associated tear of this tendon.^7^^,^^8^

The accuracy of magnetic resonance imaging (MRI) is usually lower for detection of subscapularis tears than for rotator cuff tears overall,^9^^,^^10^ with sensitivity ranging from 25% to 94%^5^^,^^11^ and specificity from 67% to 100%.^12^^,^^13^^,^^14^ Studies evaluating the diagnosis of subscapularis tears are important for clinical practice, with implications for prognosis and surgical planning. Among the published studies, some have included low magnetic field MRI,^7^^,^^13^^,^^15^^,^^16^ small samples,^15^^,^^17^ diagnosis not restricted to rotator cuff disorders,^7^^,^^12^^,^^14^^,^^15^^,^^16^^,^^18^^,^^19^^,^^20^^,^^21^ and use of intra-articular^11^^,^^14^^,^^19^ or intravenous^15^^,^^20^ contrast. These factors can generate bias in data interpretation.

## OBJECTIVE

The objective of the present study was to evaluate the accuracy of preoperative high magnetic field MRI without the use of contrast, compared with arthroscopic inspection, for identifying subscapularis tears, in cases undergoing rotator cuff repair.

## METHODS

This was a diagnostic test study comparing the findings from preoperative MRI (index test) with those from shoulder arthroscopy (reference standard) for diagnosing subscapularis tears. Operative data were collected in a standardized manner from consecutive patients between January 2013 and August 2017, by three surgeons at the same institution.

The study included patients undergoing arthroscopic surgery for rotator cuff repair who had firstly undergone preoperative 1.5-T MRI without the use of intra-articular or intravenous contrast. Patients were excluded if the interval between MRI and surgery was longer than one year, or if MRI was not available in digital format. Patients who refused to participate were also excluded, as were cases of reoperations. Examinations with moving artifacts were also excluded.

The local institutional review board approved the study (Comissão de Ética para Análise de Projetos de Pesquisa, CAPPesq), in a session on August 19, 2015, under research protocol number 12952.

### Index test - magnetic resonance imaging

All MRI scans were performed using a 1.5-T unit (HDxt, GE Medical Systems, Milwaukee, Wisconsin, United States) and a dedicated three-channel shoulder coil. The patients were placed in a supine position with their arms in a neutral position. Neither intra-articular nor intravenous gadolinium was used for any of the examinations. The protocol used was as follows: axial, oblique coronal and oblique sagittal fat-suppressed intermediate-weighted images (TR: 2717-3784 ms; TE: 42-46 ms; FOV 15 cm; slice thickness 3-4 mm; matrix 288 x 192); and oblique coronal and oblique sagittal T1-weighted images (TR: 350-517 ms; TE: minimum; FOV 14-15 cm; slice thickness 3-4 mm; matrix 288 x 192).

The examinations were blindly evaluated using Osirix 9.0 (Pixmeo SARL, Bernex, Switzerland) by two musculoskeletal (MSK) radiologists (5 and 10 years of experience) and a shoulder surgeon with 12 years of experience. The shoulder surgeon reassessed the examinations after three months, with the MRIs randomly rearranged.

### Reference standard - arthroscopy

Arthroscopic surgery was carried out with the patients placed in a beach chair position under general anesthesia and interscalene block. The integrity of the subscapularis tendon was evaluated with the 30º optic positioned in the posterior portal while an auxiliary performed the posterior lever-push maneuver.^22^ When the biceps tendon impaired visualization of the subscapularis, it was debrided or tenotomized. Arthroscopies were performed by three shoulder surgeons with 10 to 12 years of experience. The surgeons were not blinded to the MRI findings, but were blinded to the results from the study observers.

### Subscapularis evaluation

In the MRI evaluation, the subscapularis tendon was classified in one of the following categories: intact tendon, partial-thickness tear or full-thickness tear. In the arthroscopic evaluation, the same categories were used. The tendon was considered intact when no signs of tear were present, independent of presenting normal or high signals in T2. Tears were considered partial when articular, intra-substantial or longitudinal tears were present, without complete discontinuity. Full-thickness tears included those affecting the upper third, upper two-thirds or entire extent of the tendon.

### Other variables analyzed

The following clinical and demographic data were evaluated: sex; age; preoperative function, as measured on the American Shoulder and Elbow Surgeons (ASES) scale; University of California Los Angeles (UCLA) shoulder rating scale; and time between MRI and surgery. Data regarding the other tendons were collected by means of arthroscopy: supraspinatus (intact, partial tear or full-thickness tear); infraspinatus (intact or torn); and biceps (intact or torn). Biceps stability was also evaluated. For the variables visualized by means of MRI, the mean from the four evaluations of the continuous data (coracohumeral interval, measured in mm) was used. For the categorical data, a consensus reached among the evaluators regarding the following was used: fatty degeneration as described by Fuchs et al.,^23^ categorized as I or ≥ II; and presence of cysts in the lesser tuberosity, categorized as absent or present.

### Statistical analysis

Continuous data were described using means and standard deviations. Categorical data were described using absolute and proportional frequencies. Accuracy was described using the diagnosis from arthroscopy as its reference and was determined through analyses on sensitivity, specificity, positive and negative predictive value and positive and negative likelihood ratio, with their respective confidence intervals. The intra and inter-observer analyses were performed using the kappa test and the modified Fleiss kappa test, respectively. The data were presented as absolute values and were categorized in accordance with the criteria of Landis and Koch: ≥ 0.81 almost perfect; 0.61 to 0.80 substantial; 0.41 to 0.60 moderate; 0.21 to 0.40 fair; and ≤ 0.20 slight.^24^ The value set for statistical significance was ≤ 5%. The software used was SPSS^®^ for Mac 23.0 (Chicago, IL, United States). There was no need to impute data.

## RESULTS

Between January 2013 and August 2017, 411 shoulders with rotator cuff tears were operated on. The following cases were not included: 57 open repairs, 70 cases with an interval between the MRI and surgery longer than one year, 6 cases with movement artifacts, 12 cases with previous surgery and 66 cases in which MRI was not available in digital format. Thus, MRIs for 200 shoulders (195 patients) were analyzed.

Table 1 shows the general characteristics of the sample according to subscapularis tendon condition. Supraspinatus and subscapularis fatty degeneration, biceps instability, gender and age differed between the groups. The other variables did not present statistically significant differences.

**Table 1.** General sample characteristics according to the presence or absence of the different subscapularis tendon conditions

	Subscapularis tear			
	No tear (n = 61)	Partial tear (n = 83)	Full-thickness tear (n = 56)	P
Supraspinatus tear [n (%)]				
None	5 (41.7)	2 (16.7)	5 (41.7)	0.139
Partial	19 (43.2)	18 (40.9)	7 (15.9)	
Full-thickness	37 (25.7)	63 (43.8)	44 (30.6)	
Infraspinatus tear [n (%)]				
No	40 (32.3)	53 (42.7)	31 (25)	0.472
Yes (partial + full-thickness)	21 (27.6)	30 (39.5)	25 (32.9)	
Supraspinatus fatty degeneration [n (%)]**				
I	51 (32.1)	73 (45.9)	35 (22.0)	0.001*
≥ II	10 (24.4)	10 (24.4)	21 (51.2)	
Infraspinatus fatty degeneration [n (%)]**				
I	48 (33.3)	61 (42.36)	35 (24.3)	0.372
≥ II	13 (23.2)	22 (39.3)	21 (37.5)	
Subscapularis fatty degeneration [n (%)]**				
I	59 (33.1)	77 (43.3)	42 (23.6)	< 0.001*
≥ II	2 (9.1)	6 (27.3)	14 (63.3)	
LHB stability [n (%)]				
Stable	43 (41.0)	45 (44.6)	13 (12.9)	< 0.001*
Unstable	13 (16.5)	25 (46.3)	17 (31.5)	
NA	5 (29.4)	5 (29.4)	7 (41.2)	
LHB tear [n (%)]				
Not torn	34 (38.6)	34 (38.6)	20 (22.7)	0.071
Torn	27 (24.1)	49 (43.8)	36 (32.1)	
Gender [n (%)]				
Male	31 (31.3)	33 (33.3)	35 (35.4)	0.031*
Female	30 (29.7)	50 (49.5)	21 (20.8)	
Cysts in the lesser tuberosity [n (%)]				
Yes	10 (28.6)	16 (45.7)	9 (25.7)	0.855
No	51 (30.9)	67 (40.6)	47 (28.5)	
Age, years (mean ± SD)	54.97 ± 16.42	56.53 ± 10.87	58.72 ± 6.77***	0.005*
Coracohumeral interval, mm (mean ± SD)	8.19 ± 1.72	8.43 ± 2.01	8.01 ± 8.72	0.507
Time between MRI and arthroscopy, days (mean ± SD)	123.97 ± 86.68	138.01 ± 86.94	160.61 ± 106.71	0.192
ASES score (mean ± SD)	45.64 ± 21.98	43.76 ± 21.5	45.61 ± 19.05	0.608
UCLA score (mean ± SD)	15.67 ± 5.47	15.07 ± 5.44	15.66 ± 5.44	0.644

LHB = long head of the biceps; SD = standard deviation; ASES = American shoulder and elbow surgeons; UCLA = University of California Los Angeles; NA = Not applicable.

The comparison of subscapularis appearance among the five different evaluations is shown in Table 2. The shoulder surgeon detected fewer tears than those observed through arthroscopy (41.0% to 47.5% versus 69.5%). Radiologists, on the other hand, detected more tears than were observed through arthroscopy (74.5% to 78.5%). In the arthroscopic views, 30.5% of the sample presented intact tendons; 41.5%, partial tears; and 28.0%, full-thickness tears.

**Table 2.** Comparison of the integrity of the subscapularis tendon between findings from magnetic resonance imaging and arthroscopy

Subscapularis tear	Shoulder surgeon (1^st^ evaluation)		Shoulder surgeon (2^nd^ evaluation)		MSK radiologist 1		MSK radiologist 2		Arthroscopy	
	n	%	n	%	n	%	n	%	n	%
No	118	59.0%	105	52.5%	51	25.5%	43	21.5%	61	30.5%
Yes	82	41.0%	95	47.5%	149	74.5%	157	78.5%	139	69.5%
Partial	48	24.0%	67	33.5%	88	44.0%	105	52.5%	83	41.5%
Full-thickness	34	17.0%	28	14.0%	61	30.5%	52	26.0%	56	28.0%

MSK = musculoskeletal.

The inter-observer agreement was substantial for full-thickness tears (kappa 0.631; 95% confidence interval, CI 0.556-0.700; P < 0.001). For overall tears (partial or full-thickness), the results were moderate (kappa 0.463; 95% CI 0.383-0.534; P < 0.001). Intra-observer agreement was almost perfect for detection of full-thickness tears (kappa 0.809; 95% CI 0.696-0.923; P < 0.001). For overall tears, the results were moderate (kappa 0.546; 95% CI 0.430-0.662; P < 0.001). These data are shown in Table 3.

**Table 3.** Inter-observer and intra-observer reliability results

Parameter	K	95% CI		P-value
		Inferior	Superior	
Inter-observer reliability results				
Subscapularis tear (full-thickness + partial)	0.463	0.383	0.534	< 0.001
Full-thickness tear	0.631	0.556	0.700	< 0.001
Intra-observer reliability results				
Subscapularis tear (full-thickness + partial)	0.546	0.430	0.662	< 0.001
Full-thickness tear	0.809	0.696	0.923	< 0.001

CI = confidence interval.

The accuracy measurements are detailed in Table 4. The shoulder surgeon presented sensitivity of 35.7% to 39.3% (mean 37.5%) for full-thickness tears and 51.1% to 59.0% (mean 55.1%) for overall tears. The specificity was 91.7% to 94.4% (mean 93.1%) and 78.7% to 82.0% (mean 80.4%), respectively. For the MSK radiologists, the sensitivity ranged from 57.1% to 71.4% (mean 64.3%) for full-thickness tears and 83.5% to 87.1% (mean 85.3%) for overall tears, while the specificity was 85.4% to 86.1% (mean 85.8%) and 41.0% to 45.9% (mean 43.5%), respectively. Considering the average of the four evaluations, the sensitivity for overall tears was 70.2%, while the specificity was 61.9%. The accuracy of the four evaluations ranged from 77% to 81.5% (mean 78.6%) for full-thickness tears and 60.5% to 73% (mean 67.6%) for overall tears.

**Table 4.** Values relating to sensitivity, specificity, positive and negative predictive values, positive and negative likelihood ratios and accuracy of magnetic resonance imaging, compared with arthroscopy (gold-standard), for each evaluator

	Subscapularis full-thickness tear			Subscapularis tear (full-thickness + partial)		
	Mean	95% CI		Mean	95% CI	
		Inferior	Superior		Inferior	Superior
Shoulder surgeon (1^st^ evaluation)						
Sensitivity	39.3%	27.2%	52.1%	51.1%	43.2%	59.1%
Specificity	91.7%	87.5%	96.3%	82.0%	72.4%	92.3%
Positive predictive value	64.7%	49.2%	81.4%	86.6%	79.1%	94.3%
Negative predictive value	79.5%	73.1%	86.7%	42.4%	33.4%	51.7%
Accuracy	77.0%	71.2%	83.5%	60.5%	53.2%	67.3%
Shoulder surgeon (2^nd^ evaluation)						
Sensitivity	35.7%	23.2%	48.3%	59.0%	51.0%	67.1%
Specificity	94.4%	91.2%	98.5%	78.7%	68.3%	89.8%
Positive predictive value	71.4%	55.1%	88.4%	86.3%	79.7%	93.2%
Negative predictive value	79.1%	73.2%	85.2%	45.7%	36.6%	55.5%
Accuracy	78.0%	72.7%	84.4%	65.0%	58.3%	72.3%
MSK radiologist 1						
Sensitivity	71.4%	60.1%	83.4%	83.5%	77.7%	90.0%
Specificity	85.4%	80.2%	91.1%	45.9%	33.4%	58.1%
Positive predictive value	65.6%	53.1%	77.2%	77.9%	71.6%	84.7%
Negative predictive value	88.5%	83.2%	94.7%	54.9%	41.1%	69.3%
Accuracy	81.5%	76.2%	87.7%	72.0%	66.3%	78.2%
MSK radiologist 2						
Sensitivity	57.1%	44.2%	70.2%	87.1%	82.7%	93.3%
Specificity	86.1%	80.1%	92.1%	41.0%	29.5%	53.6%
Positive predictive value	61.5%	48.4%	75.3%	77.1%	70.2%	84.1%
Negative predictive value	83.8%	78.5%	90.2%	58.1%	43.7%	73.2%
Accuracy	78.0%	72.5%	84.1%	73.0%	67.3%	79.4%

CI = confidence interval; MSK = musculoskeletal.

## DISCUSSION

Subscapularis tears, which rarely occur in isolation,^5^^,^^6^ are present in the majority of arthroscopies for rotator cuff repair.^7^^,^^8^ The accuracy of magnetic resonance imaging for tear detection presents wide variation in the literature.^5^^,^^11^^,^^12^^,^^13^^,^^14^ Studies published to date have presented some weaknesses, such as use of low magnetic field MRI,^7^^,^^13^^,^^15^^,^^16^ small samples^15^^,^^17^ and evaluation of diagnoses not restricted to rotator cuff disorders.^7^^,^^11^^,^^14^^,^^15^^,^^16^^,^^18^^,^^19^^,^^20^^,^^21^ Further studies on this subject are justified, in the effort to search for more accurate results.

In the present study, the sensitivity observed regarding subscapularis tear detection ranged from 51.1% to 87.1%, with an average of 55.1% for the shoulder surgeon, 85.3% for the radiologists and 70.2% in general. Other studies evaluating 1.5-T MRI without contrast have found sensitivity values ranging from 63.6% to 82.2%.^8^^,^^21^^,^^25^

The specificity ranged from 41.0% to 82.0%, with an overall average of 61.9%. It was 80.4% for the shoulder surgeon and 43.5% for the radiologists. These values were lower than those found in other studies with the same field strength and without contrast, from which the reported values have ranged from 86.1% to 92.1%.^8^^,^^21^^,^^25^ On the other hand, they were similar to those found by Choo et al.,^18^ who used 3.0-T MRI with contrast.

Most studies in the literature have demonstrated a pattern in which specificity is superior to sensitivity with regard to detection of subscapularis tears.^5^^,^^6^^,^^7^^,^^8^^,^^12^^,^^13^^,^^16^^,^^17^^,^^21^^,^^25^^,^^26^^,^^27^^,^^28^ This pattern was also observed in an analysis on rotator cuff tears overall,^9^ but with higher percentages, thus suggesting that making the diagnosis of posterosuperior tears is less complex than that of subscapularis tears. One possible explanation for our finding is that the evaluators were aware of the objective of the study, which may have generated observation bias.

In evaluating accuracy, less pronounced differences were observed between the different observers, ranging from 60.5% to 73.0%, with an overall mean of 67.6%. It should be noted that the accuracy was higher when only full-thickness tears were analyzed, mainly due to higher specificity, ranging from 77.0% to 81.5%.

We observed an antagonistic pattern regarding the evaluations by the radiologists and the shoulder surgeon, such that sensitivity predominated in the former and specificity in the latter, but with similar accuracy. Halma et al.^29^ observed that radiologists showed greater agreement with each other, compared with orthopedists, although they did not specifically assess the subscapularis tendon.

The correlation in the intra-observer analysis was almost perfect (0.809) with regard to detection of full-thickness tears. However, in evaluating overall tears, kappa fell to a moderate level (0.546). This can be explained by the difficulty in differentiating partial tears from tendinopathy, as reported by other authors.^26^^,^^28^ To our knowledge, the present study is the first to calculate intra-observer agreement in relation to detection of subscapularis tears by means of MRI.

The inter-observer agreement found in the present study was substantial in relation to full-thickness tears (0.631), but lower than that reported by Choo et al.^18^ (0.78). Regarding overall detection of tears, considering both partial and full-thickness tears, the concordance observed in the present study was moderate (0.463), and lower than the values reported by most other authors.^16^^,^^17^^,^^19^^,^^20^^,^^26^ Only Lee et al.^20^ reported similar results. It is noteworthy that all of these authors analyzed MRI with contrast, applied intra-articularly^16^^,^^18^^,^^19^ or intravenously;^17^^,^^20^^,^^26^ and that most utilized 3.0-T devices,^17^^,^^18^^,^^20^^,^^26^ which may explain the results.

The arthroscopic inspection was performed without 70º optics, which could make it difficult to detect tears intraoperatively, according to other authors.^7^^,^^12^ In spite of this, use of standard inspection and the posterior lever-push maneuver allowed clear visualization of the subscapularis tendon in all the arthroscopies.

The inter-observer concordance analysis was performed for the two MSK radiologists and one shoulder surgeon; however, the analysis on intra-observer agreement assessed the latter only. The three evaluators knew the purpose of the study, which may have influenced the detection of tears. The surgeon had access to the MRI, both the images and the report, before performing the procedure. However, all the evaluators in the present study (MSK radiologists and shoulder surgeon) were blinded to the intraoperative findings and the surgeons were blinded to the results from the study observers.

Lastly, the time that elapsed between examinations and arthroscopy was 140 days on average, with a maximum of one year. Structural change to the tendon may have occurred during this period, although this is considered acceptable and was even used in a systematic review on this subject.^30^ Another possible criticism is that general sensitivity and specificity values were not obtained by reaching a consensus among the evaluators. However, the present authors believe that such consensuses have little practical applicability, since they are not routinely used in clinical practice.

One strong point of this study was the analysis on inter and intra-observer agreement, especially the latter. This study was the first to do this in relation to making a diagnosis of subscapularis tears. The design used, which was prospective and included consecutive cases, had only previously been used in a few articles on the same line of research.^16^^,^^27^ The large sample in the present study only involved patients undergoing arthroscopy to treat rotator cuff tears, thus increasing the internal validity of the data.

The findings from the present study have practical implications for both radiologists and orthopedists. For radiologists, they should emphasize the need for thorough evaluation of the subscapularis tendon and highlight that the differences between tears and tendinopathy may be the cause of false positives and negatives. New imaging protocols that optimize the analysis on this tendon could also be studied. For orthopedists, the findings show that cautious inspection is necessary, including actively searching for subscapularis tears, even when MRI shows no lesions. Lastly, the data presented may be useful for future meta-analyses, which would more clearly elucidate the limitations of MRI for detection of subscapularis tears.

## CONCLUSION

The 1.5-T MRI without contrast showed a mean sensitivity of 70.2% and a mean specificity of 61.9% in relation to detection of subscapularis tears. The sensitivity was higher for the MSK radiologists, while specificity was higher for the shoulder surgeon. The mean accuracy was 67.6%, which was a performance rate inferior to that for posterosuperior tears of the rotator cuff.

**Level of evidence:** Level III, Diagnostic Study.
